# A Case of Hydrothorax Successfully Treated

**Published:** 1846-06

**Authors:** 

**Affiliations:** Stark county, Ohio; Stark county, Ohio


*A Case of Hydrothorax successfully treated.* By Drs. CRUM and BRYSACHER, of Stark county, Ohio.

James Wolf, æt. 18 years, of delicate constitution, a chair-maker by occupation, had been two months under medical treatment when first seen by us. We found him extremely debilitated, with hard pulse; carotids throbbing; palpitation of the heart; anorexia; epistaxis; water in the chest, which was heard making its peculiar sound when he shifted his position. In consequence of the hydropic collection he was unable to lie down, and slept sitting propped up in bed. His feet were much swollen, and from his long confinement in his extremely emaciated condition bed sores had been formed about the sacrum and coccyx.

Reduced as was the patient we resorted to venesection. Afterwards we applied a blister over the sternum, and followed this by tartar emetic ointment to the same part. In addition, we gave him calomel, squills and gamboge, as a purgative and diuretic, and employed nitrate of potash and digitalis with a view to the reduction of arterial excitement. By a steady perseverance in this course for six weeks, the young man was relieved of all his distressing symptoms, and is now in robust health.

April 27th, 1846.

